# Fas-ligand and interleukin-6 in the cerebrospinal fluid are early predictors of hypoxic-ischemic encephalopathy and long-term outcomes after birth asphyxia in term infants

**DOI:** 10.1186/s12974-018-1253-y

**Published:** 2018-08-08

**Authors:** Kristin Leifsdottir, Huseyin Mehmet, Staffan Eksborg, Eric Herlenius

**Affiliations:** 1 Pediatric Unit, Department of Women’s and Children’s Health, Karolinska Institutet and Karolinska University Hospital, SE-171 76 Stockholm, Sweden; 2 Clinical Sciences Division, Faculty of Medicine, Imperial College London, Hammersmith Hospital Campus, Kensington, London, SW7 2AZ UK; 3 Present address: Faculty of Medicine, University of Iceland, Reykjavik, Iceland; 4 Present address: Zafgen, Inc., Boston, Massachusetts USA

**Keywords:** Asphyxia, Biomarker, Hypoxic-ischemic encephalopathy, Interleukin-6, Fas-ligand, Predictive power

## Abstract

**Background:**

Cerebral ischemia generates neuroinflammation that can induce neural cell death. This cohort study assessed whether Fas-ligand (FasL) and interleukin (IL)-6 levels in the cerebrospinal fluid (CSF) after hypoxic-ischemic encephalopathy (HIE) can serve as biomarkers of hypoxic brain injury in neonates.

**Methods:**

Term infants (> 37-week gestational age) who were admitted to the neonatal intensive care unit of Karolinska University Hospital in years 2002 to 2004 with perinatal asphyxia were enrolled prospectively. Control infants without brain pathology underwent lumbar puncture for suspected infection. FasL and IL-6 levels were measured in the CSF, by enzyme-linked immunosorbent assays. All patients underwent neurological assessment at 18 months. HIE was classified as mild, moderate, or severe (HIE I–III). Adverse neurological outcome at 18 months was defined as a mental developmental index < 85, deafness, blindness, cerebral palsy, or seizure disorder.

**Results:**

Of the 44 HIE patients, 14, 16, and 14 had HIE-I, HIE-II, and HIE-III, respectively. HIE-II and HIE-III patients had higher FasL and IL-6 levels than HIE-I patients and the 20 controls (all *p* < 0.0001). Patients with adverse outcomes had higher FasL and IL-6 levels than patients with normal outcomes and controls (both *p* < 0.0001). On receiver-operator curve analyses, FasL and IL-6 (alone and together) were highly predictive of HIE grade and outcome (areas under the curve range 0.86–0.94) and showed high sensitivity (66.7–100%). These biomarkers performed better than cord blood pH (areas under the curve: HIE grade = 0.80, adverse outcomes = 0.86).

**Conclusion:**

CSF biomarkers FasL and IL-6 predicted severity of encephalopathy and long-term outcomes in post-asphyxiated infants better than a standard biomarker.

**Electronic supplementary material:**

The online version of this article (10.1186/s12974-018-1253-y) contains supplementary material, which is available to authorized users.

## Key notes

- Following birth asphyxia, FasL and IL-6 are released into the CSF.
- FasL and IL-6 levels in CSF correlate with HIE grade and long-term clinical outcome of post-asphyxiated patients.
- Both alone and together, FasL and IL-6, accurately predict the degree of hypoxic neonatal brain injury and long-term outcomes with high sensitivity.

## Background

Hypoxic-ischemic encephalopathy (HIE) is characterized by clinical and laboratory signs of brain damage after perinatal asphyxia. It has an incidence of 2.5 per 1000 live births and associates with high rates of morbidity and mortality [1]. Cerebral hypoxic ischemia induces a strong neuroinflammatory response. After the primary insult, a cascade of events evolves to delayed cellular death that extends over several days. Apoptosis features particularly prominently in this phenomenon [2]. This biphasic pattern of neuronal death represents a window of opportunity for treatment such as therapeutic hypothermia, which is today the standard treatment for neonates with HIE [3]. However, while hypothermia is an effective treatment strategy, there is still an urgent need for additional novel interventions that protect the neurons from secondary damage and improve the clinical outcome.

The pathophysiology of HIE is not fully understood, but several studies suggest that the extrinsic apoptotic pathway involving the transmembrane death receptor Fas (CD95/Apo-1) and its natural ligand Fas-ligand (FasL) plays a central role [2, 4]. This is supported by several other studies. First, Fas receptor expression is upregulated in the brain during hypoxia, and mice that lack functional Fas receptors are protected from HIE brain injury [5]. Second, after head trauma in adults, Fas receptor and FasL are expressed in the central nervous system [6]. Third, preterm infants with post-hemorrhagic hydrocephalus have increased levels of soluble Fas receptor (sFas) in the cerebrospinal fluid (CSF), and the concentration correlates with the extent of white matter damage [7].

Evidence from experimental research indicates that inflammation and the associated production of inflammatory mediators play a significant role in the pathophysiology of brain ischemia [8]. Interleukin-6 (IL-6) is particularly interesting because its production in the post-hypoxic inflammatory cascade has been reported to have both neurotoxic and neuroprotective effects [9, 10]. The neurotoxicity of IL-6 is demonstrated by the fact that, in infants with HIE, increased IL-6 levels, in both serum and CSF, correlate positively with brain injury severity and the clinical outcome [11, 12]. The neuroprotective effect of IL-6 is exemplified by its ability to protect cerebral granular neurons from *N*-methyl-D-aspartate-induced excitotoxicity in vitro (9) and the fact that IL-6 injections into the brain after ischemia reduce ischemic brain injury (10). These opposing roles of IL-6 may reflect different functions at different stages of brain ischemia damage: specifically, it may mediate destructive inflammation during the acute phase while enhancing regeneration of the nerves in the subacute and prolonged phases [10, 13].

We hypothesized that FasL and IL-6 are upregulated in CSF samples from human infants after perinatal asphyxia and that they may correlate positively with the severity of HIE and the clinical outcome of patients. To address this, infants with HIE were compared with control infants with suspected infection in terms of FasL and IL-6 levels in the CSF.

## Methods

### Ethics

This study was performed in accordance with the tenets of the declaration of Helsinki 1975 and its revision in 1983 and European Community guidelines. The regional ethics committees at the Karolinska Institutet and the Stockholm County approved the study (Dnr 98-246, 2003-174, 2011/1891-31). Informed written consent was obtained from the parents of the enrolled patients.

### Patient population

All consecutive term infants (> 37-week gestational age) who were admitted to the neonatal intensive care unit of Karolinska University Hospital in Stockholm between October 2000 and September 2004 were enrolled prospectively into the study if they underwent clinically indicated lumbar puncture (LP), had experienced perinatal asphyxia, and met the following criteria:

1. Signs of fetal distress, as indicated by the cardiotocographic pattern of late decelerations, lack of variability, or bradycardia; meconium staining of amniotic fluid; and scalp pH < 7.1 or blood lactate levels > 4.8 mM.
2. Postnatal stress, as indicated by Apgar score < 6 at 5 min or pH ≤ 7.00/base deficit ≥ 16 mEq in the umbilical arterial/first postnatal blood from the infant, plus need for neonatal resuscitation for > 3 min.
3. Neurological signs of encephalopathy within 6 h of birth according to the NICHD classification for modified Sarnat staging [14].

Infants with congenital malformations, chromosomal abnormalities, metabolic disease, and evidence of intrauterine/perinatal infections with confirmed meningitis or with encephalopathy unrelated to birth asphyxia were excluded from the study. The control group were full-term infants who were born in the hospital in the same period and were assayed for suspected infection but whose blood and CSF were found to be negative after culture; moreover, none had any findings that were suggestive of pathology in the brain.

### Clinical assessments

All infants underwent neurological assessment shortly after birth before they were enrolled in the study. The assessment was then repeated approximately 12, 36, and 72 h and 7 days after birth and at discharge from the NICU. All assessments were conducted by the same neonatologist. HIE was classified as mild (HIE-I), moderate (HIE-II), or severe (HIE-III) according to the criteria of Sarnat and Sarnat [14].

All patients were treated under normothermic conditions and received standard treatment at the time of recruitment. This included fluid restriction, ionotropic support, and mechanical ventilation when needed as well as medical treatment for seizure activity. Continuous amplitude-integrated EEG was used to assess brain activity and suspected seizures. Given the clinical routine at the time of patient recruitment, all patients with moderate to severe encephalopathy underwent computed tomography (CT) brain scans and, in some cases, magnetic resonance imaging (MRI) on the third to fifth day of life.

All surviving patients were monitored with full neurological examinations at 3, 6, and 18 months of age that were conducted by an experienced neuropediatrician. The neurodevelopment of the patients who exhibited abnormal neurodevelopment or neurological signs on the examination at 18 months was assessed using the Bayley Scales of Infant and Toddler Development-II (BSID-II) [15], which was the Bayley version at that time. Outcome at 18 months was defined as normal outcome, adverse neurological outcome, or death. Adverse neurological outcome at 18 months was defined as a mental developmental index < 85, deafness, blindness, cerebral palsy, or seizure disorder.

The Hammersmith Infant Neurological Examination was performed on all control infants by an experienced neonatologist to get a standardized neurological assessment before discharge from NICU. Information on the outcome of the control infants at 18 months was gathered from outpatient pediatric care centers. All had normal neurological examination and none exhibited any abnormal neurological signs or history. None of the CSF samples from the controls showed indications of infection, and blood cultures were negative as well.

### CSF analysis

CSF was collected from all patients within the first 3 days of life. CSF samples were stored at − 80 °C until analyzed. The CSF concentrations of IL-6, FasL, IL-6 receptor (IL-6R), and the soluble form of the Fas receptor (sFas) were measured by enzyme-linked immunosorbent assay (Diaclone Research, Besançon, France). The detection limits were 2 pg/mL (IL-6 and IL-6R), 12 pg/mL (FasL), and 47 pg/mL (sFas). The results were normalized against total protein content, which was measured using the bicinchoninic acid assay (Pierce, Rockford, IL, USA). In some patients and controls, competition enzyme immunoassays were used to measure prostaglandin E2 metabolite (PGEM). The assay was performed according to a commercial standardized protocol (Cayman Chemicals, Ann Arbor, MI, USA). Some of the PGEM data have been presented previously [16].

### Statistical analyses

All clinical variables are presented as median (interquartile range). In terms of continuous variables, two independent groups were compared using the Mann-Whitney *U* test, while three independent groups were compared using the Kruskal-Wallis test with Dunn’s multiple comparison post hoc test. Two related groups were compared using the Wilcoxon matched-pairs signed-rank test. Three related groups were compared using the Friedman test with Dunn’s multiple comparison post hoc test. Correlations between variables were determined using the Spearman rank correlation test. In terms of categorical variables, the groups were compared using the chi-squared test. A graphical plot, namely, the receiver-operator characteristic (ROC) curve, was used to evaluate the ability of biomarkers to classify disease status and outcome. The maximum effectiveness of the biomarkers was evaluated using the Youdan Index. To evaluate the potential advantage of combining the FasL and IL-6 concentrations to identify degree of HIE and final outcome, we ranked the values of IL-6 and FasL. The rank sum was then subjected to ROC analysis.

All statistical tests were two-sided, and *p* values less than 0.05 were considered to indicate statistical significance.

## Results

### Patient characteristics at birth

In total, 46 term infants with HIE were initially enrolled into the study. Two infants with HIE who met the study eligibility criteria were then excluded because they were confirmed to have meningitis and suspected metabolic disease, respectively. Thus, 44 patients with HIE were finally included in the study. Twenty control infants without brain pathology who underwent LP for suspected infection but were then found to lack blood or CSF infection served as the control group.

The characteristics of the patient and control groups are summarized in Table 1. The two groups did not differ in terms of gestational age or birth weight. However, as expected, the HIE patients had significantly lower Apgar scores and blood gas values of pH than the control infants (both *p* < 0.001). Of the 44 patients, 14 (31.8%), 16 (36.4%), and 14 (31.8%) were classified according to the classification system of Sarnat and Sarnat as having HIE-I, HIE-II, and HIE-III in the first days of life, respectively. The three HIE groups did not differ in terms of gestational age or birth weight.

**Table 1:** Clinical data of asphyxiated newborns and newborn nonasphyxiated controls

	Controls	HIE-I	HIE-II	HIE-III
Number of patients	20	14	16	14
Gestational age (week)^a^	39.5 (38.8 to 41.7)	41.1 (37.4 to 41.3)	40.1 (38.6 to 40.3)	39.0 (37.2 to 40.4)
Birth weight (g)^a^	3614 (2820 to 4570)	3500 (3240 to 4050)	3650 (3325 to 3975)	3310 (3225 to 3595)
Gender (female:male)^a^	10:10	7:7	7:9	8:6
1-min Apgar score**	9 (7 to 9)	2 (1 to 3)	1.5 (1 to 4)	1 (1 to 2)
5-min Apgar score**	10 (8 to 10)	5 (4 to 6)	3.5 (3 to 6)	3 (0 to 4)
10-min Apgar score**	10 (10 to 10)	6.5 (6 to 7)	5 (3 to 7)	4.5 (2 to 6)
Arterial pH**	7.40 (7.13 to 7.40)	7.00 (6.90 to 7.10)	7.00 (6.80 to 7.20)	6.90 (6.70 to 7.10)
BE^a^	− 2.5 (− 18.5 to − 0)	− 17.0 (− 19.3 to − 9.4)	− 24.0 (− 27.0 to − 17.0)	− 20.0 (− 25.0 to − 14.3)
Maternal infection^a^	0	2	2	2

Data are expressed as median (IQR)

^a^Not statistically significant

**p < 0.0001

### Outcomes at 18 months

All control infants had normal outcomes at 18 months. The outcomes of the HIE patients at 18 months are summarized in Table 2. Thus, all 14 infants with HIE-I had a normal neurological examination at discharge from the NICU and a normal neurological outcome at 18 months. Of the 16 HIE-II infants, six had no neurological signs at discharge and were normal at the neurological assessment at 18 months. Two HIE-II patients had normal assessment at discharge but adverse neurological signs at 18 months. The remaining eight HIE-II patients had both neurological signs at discharge and abnormal neurological outcomes at 18 months. Thus, 10 patients with HIE-II had adverse neurological outcomes at 18 months. Of the 14 patients with HIE-III, eight died within the first 2 weeks of life of multiorgan failure due to asphyxia. The remaining six HIE-III patients all had both neurological signs at discharge and abnormal neurological outcomes at 18 months.

**Table 2:** Final outcome of asphyxiated newborns and newborn nonasphyxiated controls

	Controls	HIE-I	HIE-II	HIE-III
Number of patients	20	14	16	14
Outcome				
Normal	20	14	6	0
Adverse^a^	0	0	10	6
Death	0	0	0	8

*HIE* Hypoxic ischemic encephalopathy

^a^Adverse neurological outcome, including neurodevelopmental delay with developmental scores < 85 on BSID-III, deafness or blindness, and cerebral palsy or seizure disorder

### Association between early CT/MRI findings and outcomes at 18 months

All 30 patients with HIE-II and HIE-III underwent CT and in some cases MRI on the third to fifth day after birth. All HIE-III infants and 8 of the 10 HIE-II infants who had an adverse neurological outcome at 18 months showed signs of edema on CT. In addition, three infants with HIE-II who had a normal neurological outcome at 18 months had signs of edema on CT. Four of the HIE-III infants also underwent MRI: the other patients did not undergo MRI because it was not routinely performed at the time of recruitment. All four HIE-III patients showed profound ischemic changes in the basal ganglia and thalami. Of these, two died in the neonatal period and one survived with adverse neurological outcomes at 18 months.

### FasL and IL-6 levels in the CSF

In total, 76 CSF samples were gathered from the 44 patients and the 20 controls. One sample was obtained from 32 patients and 20 controls, and two samples were obtained from the remaining 12 patients. In the patients who underwent a single LP, the procedure was performed at a median of 22.5 (interquartile range, 15–42) h after birth and for controls 26 (13.5–48) h after birth. In the patients who underwent two LPs, the procedures were performed 14 (8–23) and 72 (60–111) h after birth, respectively. In the latter patients, the average FasL and IL-6 levels in the two CSF samples were used in the following statistical analyses.

**Fig. 1:**
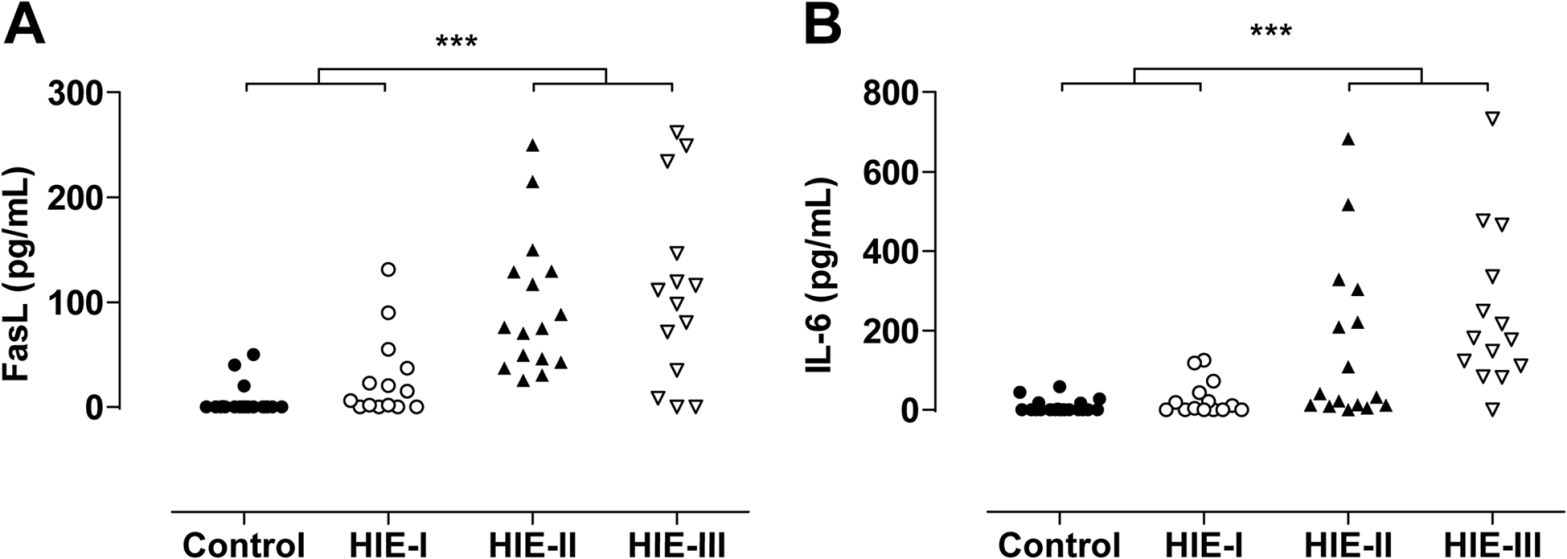
Fas-ligand (FasL) and interleukin-6 (IL-6) levels in the cerebrospinal fluid (CSF) correlate with the degree of hypoxic-ischemic encephalopathy (HIE), which was classified as mild (HIE-I, *n* = 14), moderate (HIE-II, *n* = 16), or severe (HIE-III, *n* = 14). The controls were infants with no signs of HIE (*n* = 20). FasL levels (pg/mL) (**a**) and IL-6 levels (pg/mL) (**b**) were higher in the patients with HIE-III and HIE-II than in the patients with HIE-I and the controls. ***p < 0.0001

The HIE patients had significantly higher FasL levels in the CSF (median, 62; interquartile range, 16–119 pg/mL) than the normal control infants (0, 0–0 pg/mL) (*p* < 0.0001). The patients with HIE-II (75.7, 43.7–129.4 pg/mL) and HIE-III (105.2, 28.5–168.6 pg/mL) also had significantly higher FasL levels than the patients with HIE-I (10.6, 0–41.6 pg/mL) and the controls (all *p* < 0.0001) (Fig. 1a). Difference was not found between the HIE-II and HIE-III groups.

The HIE group also had significantly higher IL-6 levels in the CSF (77, 9–214 pg/mL) than the control infants (0, 0–12.4 pg/mL) (*p* < 0.001). The patients with HIE-II (37.7, 11.4–283 pg/mL) and HIE-III (179, 104–368 pg/mL) also had higher IL-6 levels than the patients with HIE-I (6.75, 0–50.2 pg/mL) and the controls (all *p* < 0.0001) (Fig. 1b).

### Correlations between FasL and IL-6 levels with both HIE severity and outcome at 18 months

We assessed the relationship between FasL and IL-6 in the CSF and clinical outcomes. First, since Apgar score at 10 min predicts the neurological outcomes of infants with HIE [17], we assessed its relationship with the FasL and IL-6 levels in the CSF shortly after birth. FasL and IL-6 both correlated inversely with Apgar scores at 10 min (*r*_s_ = − 0.577 and − 0.622, respectively) (both *p* < 0.0001) (Additional file 1: Figure S1).

Second, correlation analyses showed that the CSF concentrations of FasL and IL-6 also correlated positively with the HIE grade (*r*_s_ = 0.6898 and 0.6864, respectively) (both *p* < 0.0001). Third, we assessed the relationship between clinical outcome at 18 months (normal or adverse neurological outcome or death) and the FasL and IL-6 levels in the CSF.

#### FasL

**Fig. 2:**
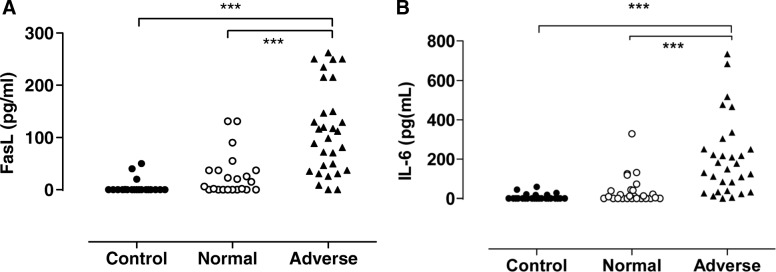
Fas-ligand (FasL) and interleukin-6 (IL-6) levels in the cerebrospinal fluid (CSF) correlate with the long-term outcomes after perinatal asphyxia. Adverse outcome is defined as adverse neurological outcome at the 18-month assessment or death. FasL levels (pg/mL) (**a**) and IL-6 (pg/mL) levels (**b**) were higher in patients with an adverse outcome (*n* = 24) than in patients with a normal outcome (*n* = 20) and the controls (*n* = 20). ***p < 0.0001

The patients with poor outcomes (i.e., adverse neurological outcome at 18 months or death) had higher FasL levels (105; 36.6–166 pg/mL) than the patients with favorable outcomes (10.6, 0–37.1 pg/mL) or the control infants (0, 0–0 pg/mL) (both *p* < 0.0001) (Fig. 2a). It should be noted, however, that the patients with adverse outcomes varied markedly in their FasL levels. Indeed, two of the patients who died had FasL levels below the detection limit. The patients who survived with adverse neurological outcomes did not differ from the patients who died in terms of CSF FasL levels. Correlation analysis showed that the CSF concentrations of FasL correlated positively with poor 18-month clinical outcomes (*r*_s_ = 0.7017) (*p* < 0.0001).

#### IL-6

The patients with poor outcomes had significantly higher IL-6 levels (162, 35.6–264 pg/mL) than the patients with normal outcomes (9.35, 0–40.8 pg/mL) or the control infants (0, 0–5.35 pg/mL) (both *p* < 0.0001) (Fig. 2b). The patients who survived with adverse neurological outcome did not differ from the patients who died in terms of CSF IL-6 levels. Correlation analyses showed that the CSF concentrations of IL-6 correlated positively with poor 18-month clinical outcomes (*r*_s_ = 0.7017) (*p* < 0.0001).

### Time-dependent trends in FasL and IL-6 levels in the CSF

**Fig. 3:**
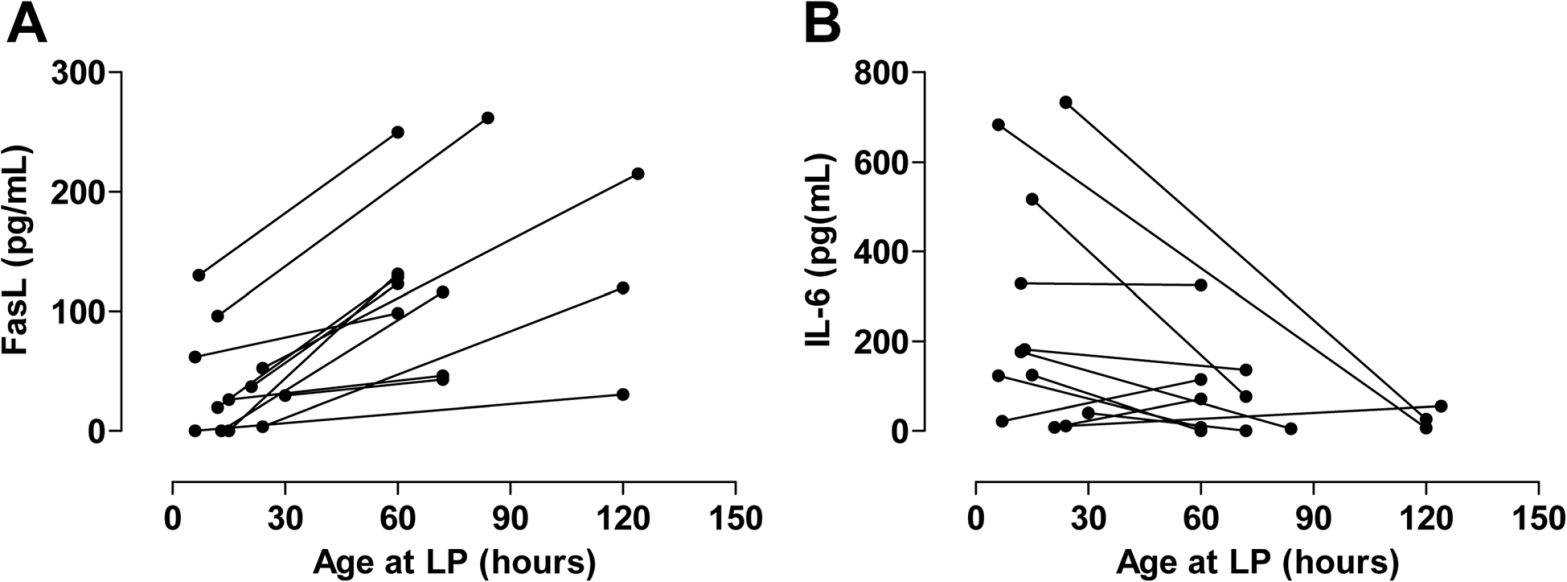
Kinetics of Fas-ligand (FasL) and interleukin-6 (IL-6) in the cerebrospinal fluid (CSF) from the 12 patients who provided a sample at two different time points. Lines indicate the samples from the same patient. **a** All patients had higher FasL levels in the later samples. **b** In 9 of the 12 patients, the IL-6 levels were higher in the earlier samples

Twelve patients provided a CSF sample at two different time points. All had HIE-II or HIE-III. Nine had adverse outcomes at 18 months. The remaining three had normal outcomes. In all patients, the FasL levels were higher in the second sample (*p* = 0.0025) (Fig. 3a).

In relation to IL-6, the levels were lower in the second sample in all but three cases (*p* = 0.0522) (Fig. 3b). In these three cases, the patients had low IL-6 levels in the first sample. Two of these three patients had normal outcomes. The nine patients who exhibited lower IL-6 levels in the second sample all had adverse 18-month outcomes. Thus, over time, FasL and IL-6 levels in the CSF rose and fell, respectively.

### FasL and IL-6 levels in the CSF are indicators for HIE severity

**Fig. 4:**
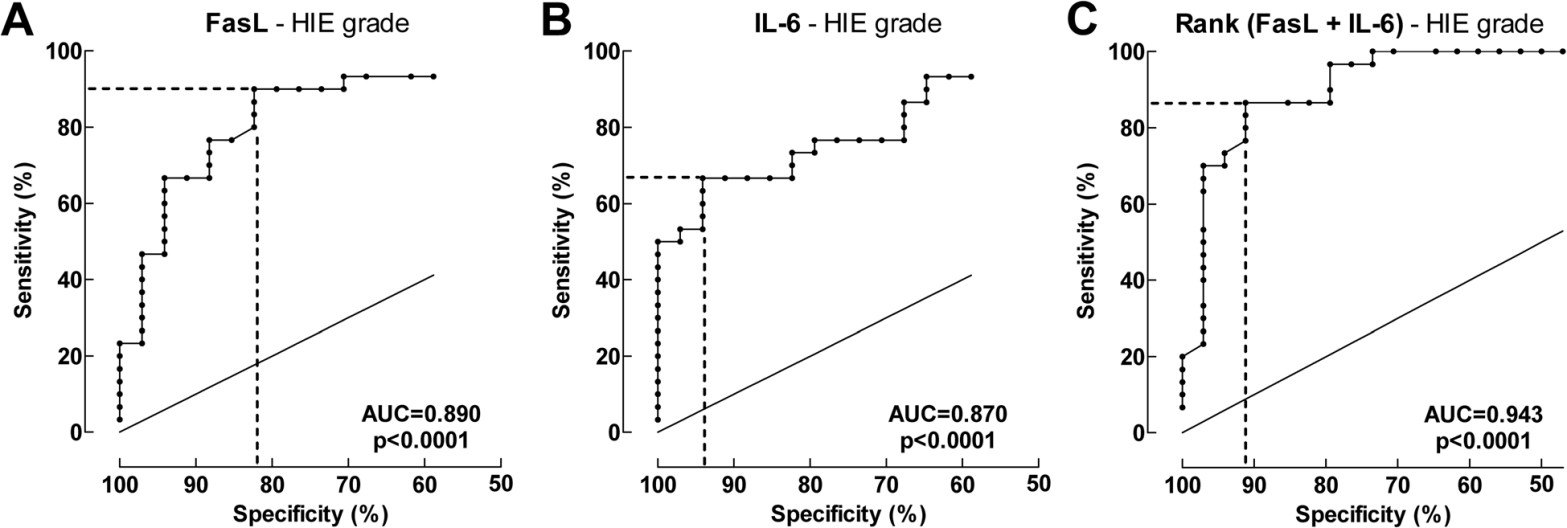
Receiver-operator characteristic (ROC) curve of Fas-ligand (FasL) and interleukin-6 (IL-6) in relation to hypoxic-ischemic encephalopathy (HIE) grade. Control and HIE I versus HIE grade II–III. **a** The AUC for FasL was 0.89. The FasL cutoff value of 24 pg/mL (dashed lines) predicted HIE grade with a sensitivity of 90.0% and a specificity of 82.4%. **b** The AUC for IL-6 was 0.87. At the cutoff value of 77 pg/mL (dashed lines), IL-6 predicted HIE grade with a sensitivity of 66.7% and a specificity of 94.1%. **c** Using a combination of ranks for IL-6 and Fas-L, the sensitivity was 86.7% and specificity 91.2% in relation to degree of HIE. The sum for ranking of IL-6 and Fas-L was used for the evaluation. The AUC for the sum for ranking was 0.94

ROC curves for FasL and IL-6 levels in relation to HIE were generated, and the areas under the ROC curves (AUCs) were estimated. These analyses showed that FasL and IL-6 predicted HIE with AUC values of 0.89 and 0.87, respectively (Fig. 4a, b). The Youdan Indices were then calculated: thus, the FasL cutoff of > 24 pg/mL predicted HIE with the highest sensitivity (90%) and specificity (82.4%), and the IL-6 cutoff of > 77 pg/mL predicted HIE with the highest sensitivity (66.7%) and specificity (94.1%).

Combining the IL-6 and FasL using rank order rendered a positive predictive value of 0.94. The sensitivity was 86.7% and specificity 91.2% in relation to degree of HIE (Fig. 4c). Notably, using the two cutoff values together also predicted HIE grade II–III with a sensitivity of 100% and a specificity of 79.4%, Table 3.

**Table 3:** Sensitivity and specificity of outcome of asphyxiated newborns and newborn nonasphyxiated controls

	HIE grade			Outcome		
	IL-6 > 77 pg/mL	FasL > 24 pg/mL	IL-6 > 77 pg/mL and/or FasL > 24 pg/mL	IL-6 > 77 pg/mL	FasL > 45 pg/mL	IL6 > 77 pg/mL and/or FasL > 45 pg/mL
True positive	20	27	30	19	19	24
True negative	32	28	27	37	33	32
False positive	2	6	7	3	7	8
False negative	10	3	0	5	5	0
Specificity (%)	94.1	82.4	79.4	92.5	82.5	80.0
Sensitivity (%)	66.7	90.0	100.0	79.2	79.2	100.0
Accuracy (%)	81.3	85.9	89.1	87.5	81.3	87.5
Negative predictive values (%)	76.2	90.3	100.0	88.1	86.8	100.0
Positive predictive values (%)	90.9	81.8	81.1	86.4	73.1	75.0

*HIE* Hypoxic ischemic encephalopathy

^a^Death or Adverse neurological outcome, including delayed neurodevelopment with mental developmental index (MDI) < 85, deafness or blindness, and cerebral palsy or seizure disorder

To determine how well FasL and IL-6 predict HIE, we also assessed the ability of cord blood pH, which is the established biomarker for perinatal asphyxia, to predict HIE. Cord blood pH predicted HIE with an AUC of 0.80. Cord blood pH < 6.85 predicted HIE with a sensitivity of 66.7% and a specificity of 80.0% (data not shown). Thus, FasL and IL-6 alone and together predicted the degree of HIE better than this well-known standard marker of perinatal asphyxia.

### Ability of FasL and IL-6 levels in the CSF to predict adverse outcomes at 18 months

**Fig. 5:**
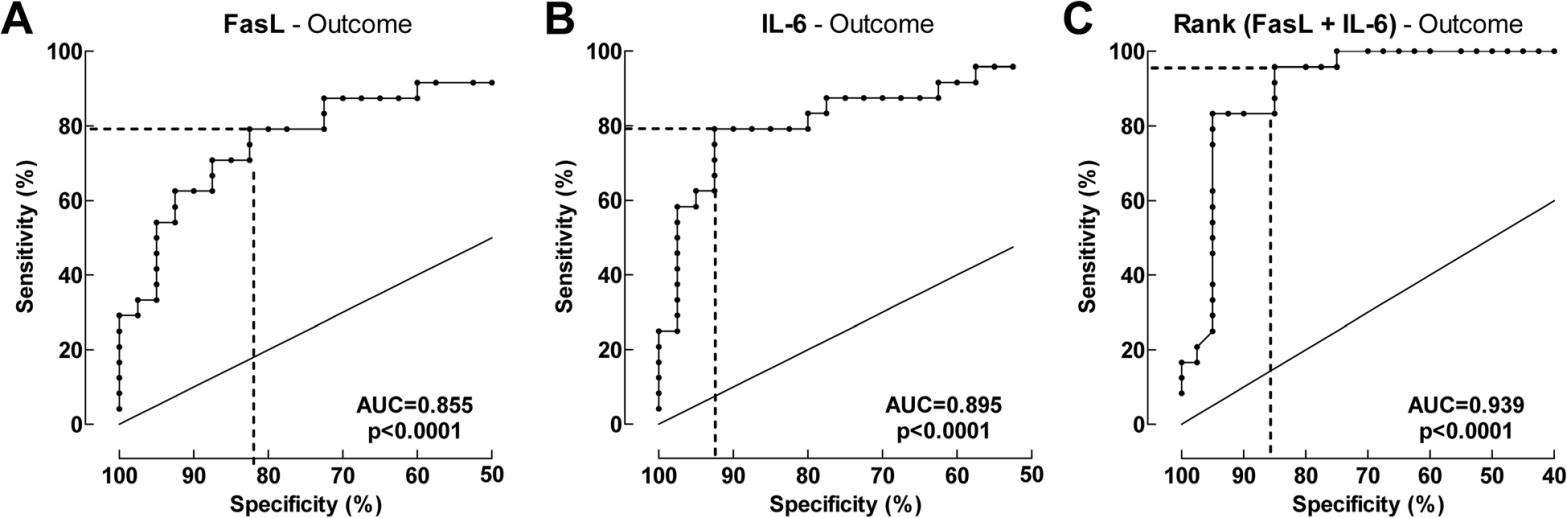
Receiver-operator characteristic (ROC) curve of Fas-ligand (FasL) and interleukin-6 (IL-6) in relation to adverse outcome. Adverse outcome is defined as adverse neurological outcome at the 18-month assessment or death. **a** The AUC for FasL was 0.86. The FasL cutoff value of 45 pg/mL (dashed lines) predicted adverse outcomes with a sensitivity of 79.2% and a specificity of 82.5%. **b** The AUC for IL-6 was 0.90. The IL-6 cutoff value of 77 pg/mL (dashed lines) predicted adverse outcomes with a sensitivity of 79.2% and a specificity of 92.5%. **c** Using a combination of ranks for IL-6 and Fas-L, the sensitivity was 95.8% and specificity 85.7% in relation to outcome. The sum for ranking of IL-6 and Fas-L was used for the evaluation. The AUC for the sum for ranking was 0.94

ROC curves for FasL and IL-6 levels in relation to the outcomes at 18 months were generated, and the AUCs were estimated. These analyses showed that FasL and IL-6 predicted adverse 18-month outcomes (i.e., adverse neurological outcome or death) with AUCs of 0.86 and 0.90, respectively (Fig. 5a, b). The Youdan Indices were calculated: the FasL cutoff of > 45 pg/mL predicted adverse outcomes with a sensitivity of 79.2% and a specificity of 82.5%, and the IL-6 cutoff of > 77 pg/mL predicted adverse outcomes with a sensitivity of 79.2% and a specificity of 92.5%.

Combining the IL-6 and FasL using rank order rendered a positive predictive value of 0.94**.** The sensitivity was 95.8% and specificity 85.7% in relation to outcome (Fig. 5c). Notably, using the two cutoff values together also predicted adverse outcome with a sensitivity of 100% and a specificity of 80.0% (Table 3).

By contrast, cord blood pH predicted adverse outcomes with an AUC of 0.86. Cord blood pH < 6.85 predicted adverse outcomes with a sensitivity of 66.7% and a specificity of 93.6% (data not shown). Thus, FasL and IL-6 alone and together predicted adverse outcomes at 18 months better than this well-known standard marker of perinatal asphyxia.

### Soluble forms of FasL and IL-6 receptors in the CSF

Our findings prompted us to determine the levels of the soluble forms of the receptors for FasL and IL-6. sFas levels were measured in 26 patient samples and 20 control samples. Seven patient samples had high sFas levels (ranging from 355 to 1160 pg/mL), and all but one of these patients had an adverse outcome at 18 months. The median sFas concentration of the patients was 441 pg/mL. By contrast, sFas levels in all control infant samples were below the limits of detection (Additional file 2: Table S1).

All patient CSF samples had significant amounts (i.e., above the upper limit of the test) of IL-6R. By contrast, the IL-6R levels in all control infant samples were below the limits of detection (data not shown).

### Relationship between PGEM and IL-6 levels in the CSF

**Fig. 6:**
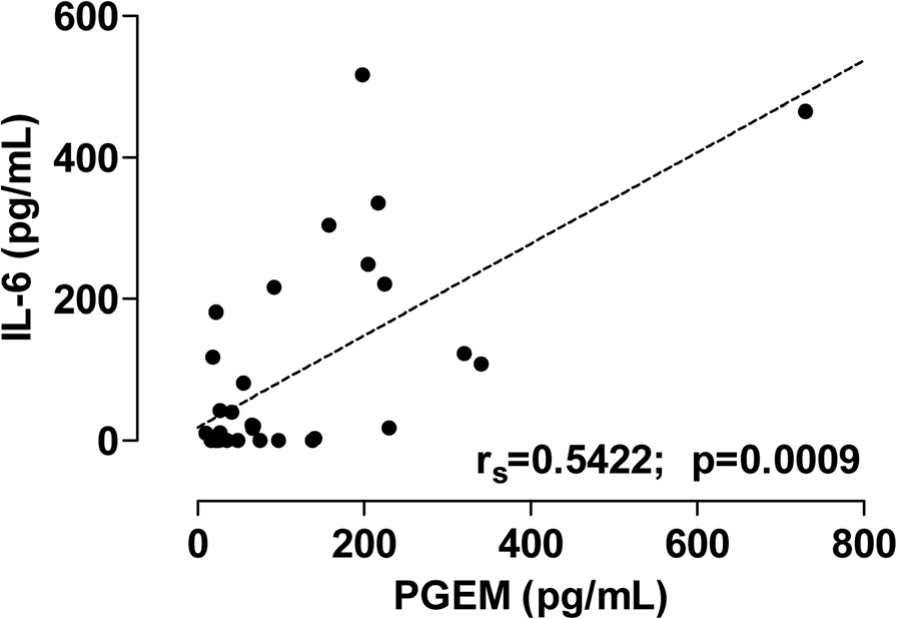
Levels of interleukin-6 (IL-6) and the prostaglandin E2 metabolite (PGEM) in the cerebrospinal fluid (CSF) correlate (*p* = 0.0009). *r*_s_ Spearman rank correlation coefficient

We reported previously that increased levels of PGEM in the CSF correlate with 18-month clinical outcomes of infants with HIE [16]. We measured the PGEM levels in the CSF of 25 patients and nine controls in this study. We found that the PGEM levels correlated significantly with the IL-6 levels in the CSF (*p* = 0.0009) (Fig. 6). In contrast, no correlation was found between the FasL levels and the PGEM levels.

The inclusion of PGEM data in addition to IL-6 and FasL data in Table 3 increased neither specificity nor sensitivity for identification of patients with HIE grade II–III or poor outcome.

## Discussion

The main findings of this study were that post-asphyxiated neonates had elevated levels of the inflammatory mediators IL-6 and FasL in their CSF shortly after birth. Since the levels of both molecules correlated positively with HIE grade and poor 18-month clinical outcome, they may be useful as biomarkers for the severity of hypoxic brain injury and for predicting the long-term outcome after perinatal asphyxia.

Animal and human studies show that a variety of cytokines are expressed in the brain in cerebral ischemia and that the cytokine profile associates significantly with the severity of ischemic brain damage [18, 19]. Moreover, it has been suggested that cytokines can either induce or ameliorate ischemic brain injury and that these opposing functions depend on both the phase of the neural cell damage and the severity of the insult [10]. These time- and severity-dependent changes in cytokine levels mean that the levels of a given cytokine in a CSF sample will only provide a snapshot of an ongoing process; it is often difficult to know when exactly a cytokine is promoting ischemic brain injury or acting protectively. This is exemplified by several studies on the role of IL-6 in therapeutic hypothermia, which is thought to be neuroprotective because it has anti-inflammatory properties [20]. When Jenkins and colleagues serially measured cytokines every 12 h for 4 days after the birth, they found that HIE patients who underwent hypothermia treatment had higher serum IL-6 levels at all time points than HIE infants who were treated under normothermic conditions [13]. Moreover, the IL-6 levels in the hypothermia-treated group were biphasic while the IL-6 levels in the normothermic patients tended to decline over time. Significantly, while high IL-6 levels early after the insult, in both groups, are associated with adverse 18-month outcomes, a secondary peak of IL-6 is associated with better outcomes [13, 21]. This suggests that IL-6 may have biphasic roles in the pathogenesis of hypoxic brain injury: it induces inflammation and injury early after the insult but then contributes to cytokine-mediated repair at later time points [21, 22].

In the present study, the patients did not receive therapeutic hypothermia because it was not an established therapy for HIE at the time of recruitment. We found that, in the 12 patients who underwent LP at two separate time points after birth, the IL-6 levels were lower in the second sample in all but three cases (Fig. 3b). This is consistent with previous observations, regarding normothermic patients where serum IL-6 levels after birth asphyxia were characterized in [13, 21]. All patients in the present study who exhibited a drop in IL-6 levels had adverse 18-month outcomes. Two of the three patients who had increased IL-6 levels between first and second LP had normal outcomes. However, those patients had low IL-6 levels in the first sample and the rise was small (9.9 pg/mL, 9–10.5 in LP1 and 63.3 pg/mL, 59.5–67 in LP2) (Fig. 3b).

It should be noted that we measured IL-6 levels in the CSF, whereas several studies measure the serum IL-6 levels, e.g., [13, 21]. The fact that our findings closely resemble those of normothermic patients [13] probably reflects the fact that the serum and CSF concentrations of IL-6 in term infants with asphyxia correlate [23].

A systematic review of potential brain injury biomarkers identified serum IL-6 as one of the few independent predictors of adverse outcome in survivors of HIE [24]. However, CSF IL-6 may be a better predictor of adverse outcomes because local cytokine profiles are often poorly reflected in the plasma: this is because the plasma levels can also be shaped by secondary reactions to the injury [25, 26].

Intracellular cytokine levels, which closely reflect cytokine production at a cellular level and show more stable kinetics in time, have recently been analyzed in term infants requiring systemic hypothermia after perinatal asphyxia [27]. In that study, intracellular IL-6 levels in CD4+ peripheral blood mononuclear cells peaked at 24 h post-asphyxia. However, IL-6 levels exhibited a large variability and did not differ between the two patient groups moderate (*n* = 17) and severe (*n* = 11) asphyxia. Thus, IL-6 plays an important role in the initial inflammatory response, but determination of CSF levels of IL-6 might better predict adverse outcomes in survivors of HIE [28].

IL-6 initiates a signal transduction cascade by binding to specific IL-6 membrane receptors (IL-6R). Apoptosis associates with the shedding of membrane components, including IL-6R, which then facilitates IL-6 signaling in neighboring cells [29]. While most brain cells are not responsive to IL-6 alone, they can be stimulated by IL-6 bound to a soluble form of IL-6 receptor in a process called trans-signal activation [30]. The present study showed that the HIE patients had high concentrations of IL-6R in all samples whereas the IL-6R levels in all control infants were below the detection limit. It is possible that the high levels of IL-6R in the CSF of the HIE patients reflect an apoptotic process that is taking place in the brain at the time the samples were gathered. Unfortunately, the CSF samples were limited in volume, which meant that the IL-6R measurements were not accurate enough to assess the relationship between IL-6R levels and long-term outcomes.

This study showed that FasL, which plays a key role in apoptosis, also correlated positively with both the HIE grade (Fig. 1a) and the adverse long-term outcomes (Fig. 2a) in HIE patients. Experimental studies suggest that hypothermia blocks the Fas-mediated intrinsic and extrinsic apoptosis pathways [31, 32]; however, clinical studies are needed to confirm this. The naturally occurring soluble form of Fas receptor, sFas, prevents cell ligation with FasL, and this blocking of FasL inhibits neuronal cell apoptosis in experimental brain ischemia [33]. sFas has been found in the CSF from neonates with hydrocephalus [34], but in that study, FasL was not detected. We found that, while none of the control infants had sFas in their CSF, 7 of 26 HIE patients had high levels and that all but one of these had adverse outcomes at 18 months. This suggests that CSF sFas may not actually protect HIE infants from FasL-induced apoptosis and the consequent poor outcomes.

Prostaglandin E2 (PGE2) is another important mediator of neuroinflammation [35]. PGE2 and its derivative PGEM rapidly increase during hypoxia [36]. We found that IL-6 levels in the CSF correlated positively with the PGEM levels in the CSF (Fig. 6). However, since only a subgroup of the patient cohort was examined for both PGEM and IL-6, and we have already established that increased levels of PGEM in CSF correlate positively with the clinical outcome of infants with HIE [16], we thus focused our further analyses on FasL and IL-6.

Our ROC curve analyses showed that IL-6 and FasL in the CSF may be useful as additional tools for evaluating the severity of hypoxic-ischemic encephalopathy in post-asphyxiated infants and for predicting the long-term outcomes. First, this study showed that FasL (AUC = 0.89) and IL-6 (AUC = 0.87) predict HIE grade even better than cord blood pH (AUC = 0.80). When FasL and IL-6 were combined, they predicted HIE grade particularly well (AUC = 0.94). Second, FasL (AUC = 0.90) and IL-6 (AUC = 0.86) predicted adverse outcomes better than cord blood pH (0.86). This performance was even better when FasL and IL-6 were combined (AUC = 0.94).

Notably, this novel and unprecedented results of prediction for HIE and long-term outcome, using CSF biomarkers, could help guide treatment decisions. Thus, prospective studies concerning the benefits of CSF cytokine measurements also in hypothermic treated patients would indeed be of value.

The main limitation of this study was the time lapse between patient recruitment and the presentation of the results. However, the fact that the patients were recruited before hypothermia became the standard treatment for HIE could be considered an advantage because it allowed us to compare our FasL and IL-6 findings in that setting with the findings of other studies, which were all conducted after therapeutic hypothermia was introduced as a standard treatment. Another limitation is that, while an experienced pediatric neurologist performed the routine clinical follow-up on all post-asphyxiated children at the Karolinska Hospital at that time, only the patients with neurological symptoms or signs at 18 months were assessed with the Bayley developmental evaluation assessment: the patients without signs did not undergo BSID-II scoring. Further studies on the diagnostic benefits of monitoring FasL and IL-6, with or without other biomarkers, in newborn infants with HIE who are treated with therapeutic hypothermia are warranted. Further studies on these biomarkers may also shed more light on the pathology of brain damage and determine whether targeting them may be of therapeutic benefit.

## Conclusion

IL-6 and FasL are released into the CSF during birth asphyxia. These biomarkers support the clinical diagnosis of HIE degree, thus correlating to the degree of brain injury, and are useful for predicting the long-term clinical outcome. Thus, they may aid the early decision-making regarding treatment of asphyxiated newborns.

## Additional files

Additional file 1: **Figure S1.** Fas-ligand (FasL) (A) and Interleukin-6 (IL-6) (B) levels in the cerebrospinal fluid (CSF) correlate inversely with Apgar scores at 10 min (*r*_s_ = − 0.577 and − 0.622, respectively. *r*_s_ = Spearman rank correlation coefficient) (both *p* < 0.0001). (TIF 119 kb)

Additional file 2: **Table S1.** Soluble Fas receptor (sFas) levels in 26 asphyxia patients and 20 controls. (DOCX 15 kb)

## Abbreviations

AUC: Area Under the ROC curve
BSID-II: Bayley Scales of Infant and Toddler Development-II
CSF: Cerebrospinal fluid
CT: Computed tomography
FasL: Fas-ligand
HIE: Hypoxic-ischemic encephalopathy
IL-6: Interleukin (IL)-6
IL-6R: IL-6 receptor
LP: Lumbar puncture
MRI: Magnetic resonance imaging
PGE2: Prostaglandin E2
PGEM: Prostaglandin E2 metabolite
ROC: Receiver-operator characteristic
sFas: Soluble form of Fas receptor
