# Supplementary material for: Fas-ligand and interleukin-6 in the cerebrospinal fluid are early predictors of hypoxic-ischemic encephalopathy and long-term outcomes after birth asphyxia in term infants
Source: J Neuroinflammation. 2018 Aug 8;15:223. doi: 10.1186/s12974-018-1253-y (PMC6083505; doi:10.1186/s12974-018-1253-y)
Supplement: Supplementary file 2 — Table S1. Soluble Fas receptor (sFas) levels in 26 asphyxia patients and 20 controls. (DOCX 15 kb) [file 12974_2018_1253_MOESM2_ESM.docx]

| **Table S1. CSF samples with sFas values above detection limit** | | | | |  |
| --- | --- | --- | --- | --- | --- |
| **Patients (n=7)** | **HIE grade** | **Outcome** | **sFas** | **FasL** | **IL-6** |
|  |  |  |  |  |  |
| 1. | III | Died | 514,5* | 0* | 476,3* |
| 2. | II | Adverse† | 1160* | 0* | 687,6* |
| 3. | III | Adverse† | 355* | 116* | 22* |
| 4. | III | Adverse† | 637,7* | 237* | 114,3* |
| 5. | II | Adverse† | 1065,2* | 42,2* | 28* |
| 6. | II | Adverse† | 398,5* | 215,3* | 55,5* |
| 7. | I | Normal | 362* | 0* | 0* |
| *pg/mL |  |  |  |  |  |

†Adverse neurological outcome

In all other examined patient samples from asphyxiated patients (n=19), including all controls (n=20), sFas-levels were below the limit of detection.
